# How daily supervisor abuse and coworker support affect daily work engagement

**DOI:** 10.3389/fpsyg.2022.880528

**Published:** 2022-07-22

**Authors:** Hongqing Wang, Tianzhen Tang

**Affiliations:** ^1^School of Business, Nanjing Audit University, Nanjing, China; ^2^School of Business, Nanjing University, Nanjing, China

**Keywords:** abusive supervision, work engagement, coworker support, daily diary study, job demands resources model

## Abstract

The purpose of this study was to explore the dynamic and intervention mechanisms of daily abusive experience affecting daily work engagement. Drawing on conservation of resources (COR) theory, we examine the effect of daily abusive supervision on daily work engagement through daily negative emotions from the resource consumption perspective, and the moderation effect of coworker support from the resource provision perspective. Using a daily diary approach and based on a sample of 73 employees for 5 consecutive days in China. The results reveal that daily abusive supervision has a significant negative effect on daily work engagement, daily negative emotions mediate this relationship, and coworker support had a cross-level moderating effect between daily abusive supervision and daily negative emotions. Our study shows ways to boost employees’ daily work engagement and especially ways buffer the negative effect of abused experience on work engagement.

## Introduction

Work engagement plays a vital role in fostering numerous desirable work behaviors (Arshad et al., 2021), such as job resourcefulness, life satisfaction, task and contextual performance, career satisfaction, organizational citizenship behaviors, creativity, subjective career success, flourishing at work, employee wellbeing, and organizational effectiveness (Ariza-Montes et al., 2018; Chen, 2019; Grobelna, 2019; Aggarwal et al., 2020; Chen et al., 2021; Herr et al., 2021; Weiss and Zacher, 2022). In other words, work engagement is crucial for organizations seeking to improve labor efficiency and attain a competitive advantage (Kulikowski and Sedlak, 2017; Huang et al., 2022). However, there is currently a “worldwide employee engagement crisis,” as only 13% of employees working for organizations are engaged (Mann and Harter, 2016; Rafiq et al., 2019). Therefore, determining how to foster employees’ work engagement has been increasingly studied by organizational researchers and practitioners.

As the provider of job resources, leadership behavior is a fundamental factor in determining employees’ work engagement, and 70% of the variance in work engagement can be explained by leadership behavior, such as service leadership, self-leadership, paradoxical leadership, transformational leadership, authentic leadership, engaging leadership and empowering leadership (Cai et al., 2018; Kaya and Karatepe, 2020; Zheng et al., 2020; Dorssen et al., 2021; Fürstenberg et al., 2021; Tuin et al., 2021). However, leaders may not only provide resources for employees through positive leadership behaviors, but also consume employees’ resources through destructive behaviors. As a typical form of destructive leadership, abusive supervision has been widely studied by organizational scholars, due to the presence of high power distance, abusive supervision is more common in the Chinese cultural context. Employees tend to see their supervisors as one of their greatest sources of resources, and abusive supervision might be a key source of resource loss (Hsu et al., 2021). Given the improbability of eliminating abusive supervision from organizational contexts (Kirrane et al., 2018), it is more meaningful to explore how abusive supervision affects work engagement and how to mitigate its negative effects (Huh and Lee, 2021).

Based on conservation of resources (COR) theory, abusive supervision has been conceived as the most important stressor in the workplace that may deplete employees’ available emotional resources because their supervisors are unsupportive (Wang et al., 2020) and elicit employees’ negative emotions, which in turn diminish employees’ work engagement because negative emotions lead to a loss of physical and emotional resources that are necessary to sustain vitality, enthusiasm and concentration (Wang and Shi, 2020). COR theory holds that social support, among the job resources available in the workplace, plays a significant role in the buffering process enabling employees to deal with the detrimental influences of job stressors (Huh and Lee, 2021). Coworkers and leaders are the main sources of support for employees in an organization, since sources of support may need to be independent of sources of stressors because support from the same source that is provoking a stressor might be awkward and unproductive (Mayo et al., 2012). In addition, employees spend more time with their coworkers, and thus, it is very important to explore the effect of coworker support on abused employees’ reactions.

Previous studies have generally conceptualized work engagement as a stable individual trait, while recent empirical evidence has shown that work engagement not only shows differences between individuals and can operate as a trait variable but also varies within a person over time and should be examined as a state variable; approximately one-third of the total variance in work engagement can be explained by within-person variation (Garrick et al., 2012; Kühnel et al., 2012). Researchers have found that abusive supervision is also a state variable; that is, supervisors exhibit more within-person than between-person variation in abusive behavior (Barnes et al., 2015). Although revealing the within-person predictor of work engagement is useful for management practices, previous studies have paid insufficient attention to the effect of daily leadership behavior on work engagement (Kühnel et al., 2012).

**FIGURE 1:**
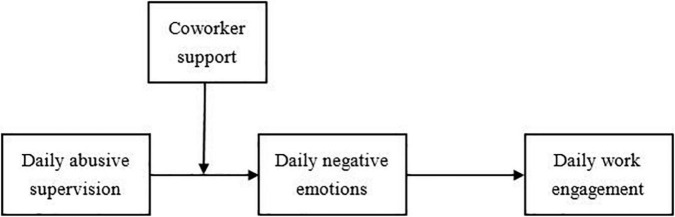
Theoretical framework.

As stated above, at the within-person level, we explore the mediating mechanism of leaders’ daily abusive supervision on employees’ daily work engagement through negative emotions. At the between-person level, we explore the cross-level moderating effect of coworker support between daily abusive supervision and daily negative emotions (see Figure 1). In sum, the contributions of this study to the literature are as follows. First, despite a recent increase in the literature on the use of job resources to mitigate the negative effects of abusive supervision, support regarding how increased work engagement, can be promoted under abusive supervision is scarce (Tepper, 2007; Khan et al., 2022). From the perspective of job resources, we reveal ways to improve the work engagement of employees who suffer from abusive supervision. Second, many studies on abusive supervision and work engagement have focused on between-person differences and ignored within-person differences. We explore the dynamic mechanism of daily abusive supervision on daily work engagement using a within-person approach, which is much more realistic than a between-person approach that considers all behavior occurring on different days as a whole and only examines averages values (Breevaart et al., 2016).

## Literature review and hypothesis development

### Daily abusive supervision and work engagement

Abusive supervision behaviors refer to any display of hostile verbal and non-verbal behavior, excluding physical contact. These behaviors are likely to vary on a day-to-day basis and can involve ridiculing subordinates in front of others; withholding important information; and using disparaging language, threats, and intimidation tactics (Barnes et al., 2015, p. 1420). COR theory proposes that when individuals face the threat of resource loss, people always seek to maintain existing resources or acquire new ones (Hobfoll and Shirom, 2001). When employees face abusive behavior from their leaders, they lose some coveted resources, such as status, position, self-esteem and energy (Mayo et al., 2012), and they cannot obtain more job resources from their leaders, such as information and encouragement. In addition, they need to spend more resources to deal with their abusive experience. In details, in order to avoid further the occurrence of abusive behavior, they need to spend more time and effort on their relationship with their leader (Arshad et al., 2021). Work engagement is a transient, positive, fulfilling, and work-related state of mind that fluctuates within individuals over a short period of time and is characterized by vigor, dedication, and absorption (Breevaart et al., 2014, p. 140). Vigor reflects a state of high energy at work, dedication refers to strong identification with enthusiasm for one’s work, and absorption is characterized by being completely immersed in one’s work (Schaufeli et al., 2002). These active states require abundant energy, which is a form of expression of high resources (Wang and Shi, 2020). As stated above, on the one hand, leader abusive behavior may result in abused employees objectively not having the adequate resources to devote to work engagement. On the other hand, abused employees are more likely to subjectively withhold work engagement for the sake of preserving remaining resources or avoiding a further depletion of resources (Ampofo, 2020). Bakker (2014) found that work engagement fluctuates on a daily basis and that this daily fluctuation is driven in part by negative employee experiences at work. Therefore, abusive supervision is negatively related to employee engagement.

H1. Daily abusive supervision is negatively related to daily work engagement.

### The mediating role of daily negative mood

Behavior in organizations is intrinsically driven by members’ emotional reactions to events in their environment; in fact, emotions play a central role in human behavior in general (Ashkanasy et al., 2017). According to COR theory, negative events in the workplace, such as being a victim of hostile behaviors or being exposed to negative interpersonal conflicts (Michel et al., 2016), may induce employees’ negative emotional responses (Weiss and Cropanzano, 1996). First, these negative events will cause employees to perceive the threat of resource loss. In this case, on the one hand, individuals may have a series of emergency responses to protect and restore their resources, such as emotional responses; on the other hand, employees may trigger negative emotions because they fear that they do not have enough resources to cope with negative events (Wang and Shi, 2020). Second, these unfair interpersonal interactions lead employees to predict that they will not receive a return on their investment of resources, which in turn elicits a negative emotional reaction (Wang et al., 2022). Abusive leaders display hostile verbal and/or non-verbal behaviors toward their subordinates, such as ridiculing or publicly criticizing subordinates and making aggressive eye contact (Tepper, 2000). In the case of abusive supervision, it can be expected that employees will appraise it as negative and unfair treatment; under the pressure of protecting existing resources and avoiding further depletion of resources, employees are prone to develop negative emotions or work-related affect related to abuse (Michel et al., 2016). Extant research has confirmed that when faced with such situations, subordinates who perceive that they have been subjected to abusive behavior may be more likely to experience a range of emotions including shame, anger, fear, anxiety, hostility (Peng et al., 2019; Korman et al., 2021).

According to COR theory, people’s resources are threatened or lost after negative events, and they may experience negative emotions when they try to deal with primary resource loss. However, negative emotions also function as resource consumption, and they may trigger secondary resource loss; therefore, to avoid a further depletion of resources, they may engage in ineffective behaviors (Subramony et al., 2021; Thompson et al., 2022). In summary, one’s emotional response to workplace events largely determines one’s attitudes and subsequent behaviors (Carlson et al., 2011). As stated above, abusive supervision, as a negative workplace event, may trigger negative emotions. Negative emotions signal a threat to one’s personal resources; when individuals focus on the source and coping strategies of negative emotions, their cognitive load may increase, which may then result in the loss of their cognitive and emotional resources (Beal et al., 2005; Greenidge and Coyne, 2014; Wang and Shi, 2020). Work engagement requires employees to direct all of their attention toward organizational goals, to feel connected to their work, and to believe that they can deal with their job’s demands; it denotes an active and positive work-related state characterized by a commitment to and mental involvement with work tasks (Swati, 2016). When a person is in a negative emotional state, their attention diverts from the work task to protect resources from further loss and subsequently leads to avoidance behavior and disengagement (Elliot, 2006) because these negative behaviors may help the victim feel better as a result of conserving their resources (Matta et al., 2014). Thus, negative emotions may cause an off-task focus and lead to a failure to provide the vitality, enthusiasm and concentration that are necessary for sustaining work engagement (Kuba and Scheibe, 2017), which aligns with Gkorezis et al.’s (2016) finding that negative emotions experience on Monday morning will be negatively associated with engagement during work on Monday.

H2. Daily negative emotion mediates the relationship between daily abusive supervision and daily work engagement.

### The cross-level moderating role of coworker support

Conservation of resources theory holds that social support is an important resource for individuals having to cope with stressors, since it can both increase one’s resource pool and can replace the resources that one lacks (Hobfoll, 1989). Coworker and supervisor support have long been identified as two important job resources that help employees deal with stressors at work (Kim et al., 2017). When employees experience abusive behavior from their supervisors, they need more coworker support because under these situations, abused employees may feel inconsistent and insincere even when the abusive supervisor is willing to offer them support (Mayo et al., 2012). Coworker support “refers to employees’ beliefs about the extent to which coworkers provide them with desirable resources in the form of emotional support (e.g., showing concern) and instrumental assistance (e.g., helping with work tasks)” (Poon, 2011, p. 67). These beliefs contribute to achieving work goals, gaining humanistic care, fostering personal development, and reducing job stress (Bakker and Demerouti, 2007). Therefore, coworker support may buffer the effect of abusive supervision on negative emotions. First, coworkers may act as good listeners for abused employees which could help them vent their negative emotions effectively. Second, when abused employees share their abusive experiences with coworkers, coworkers may demonstrate sympathy, understanding and concern and friendly, warm relations. On the one hand, this support fulfills abused employees’ needs for esteem, approval, and affiliation and then alleviates their negative emotional experiences with the organization (Tafvelin et al., 2019); on the other hand, these emotional resources can make up for the loss of resources caused by abusive experiences, leaving employees with sufficient resources to regulate their negative emotions (Singh et al., 2019). Third, coworkers can share effective measures for abused employees to cope with leaders’ abusive behaviors or provide new ideas that can help employees to develop new coping mechanisms (Mayo et al., 2012). These informational resources may relieve the negative emotions generated by abusive experiences.

H3. Coworker support moderates the relationship between daily abusive supervision and daily negative emotion.

## Materials and methods

### Participants and procedure

We used a daily diary survey to collect data. The survey included two questionnaires. Questionnaire A was mainly used to collect between-person variables, which included demographic variables and coworker support. Questionnaire B was mainly used to collect within-person variables, which included daily abusive supervision, daily negative emotions and daily work engagement. Consistent with previous studies, questionnaire A needed to be completed only once; questionnaire B was completed for 1 week, and participants completed the questionnaire once a day. The participants were mainly frontline employees of Chinese chain restaurants. The questionnaires were mainly conducted on site. We offered payment to increase participation: participants could obtain 10 yuan when they completed questionnaire B once, but they had to complete the questionnaire for 5 days before they were paid a total of 50 yuan. To match the questionnaires, in the first survey, we assigned the participants questionnaires marked with a code and asked them to remember their code. These codes were used to track each participant’s payment, so that when he or she completed the questionnaires for the remaining 4 days, each participant could write this code on his or her questionnaires.

The final sample of participants who provided daily diary data for all 5 days consisted of 73 employees, which yielded a within-person sample of 365 responses. Among the 73 participants, 74% were men, and 26% were women. In terms of education, 15.1% participants were at the high school level, 41.1% were at the junior college level, 35.3% were at the undergraduate level, and 8.2% were at the graduate level or above. In terms of age, 43.8% participants were under 20 years old, 47.9% were aged 21 to 25, and 8.3% were older than 26 years old. The respondents were young on average because restaurant waiters in China tend to be young.

### Measures

Since the participants were asked to complete the within-person questionnaire for 5 days, and they may feel burnout, so we measured all day-level variables using shortened versions of existing scales. Consistent with extant research, we chose three to five items for each day-level variable that had the highest loadings and could be administered on a daily basis. The between-person variables were measured using the original scales. All of the variables were measured on a 7-point Likert scale (1 = strongly disagree, 7 = strongly agree).

#### Daily abusive supervision

Abusive supervision, a day-level variable, was measured using a shortened scale with five items, which were derived from Tepper’s (2000) scale. Example items include the following: “Today, my supervisor put me down in front of others” and “Today, my supervisor was rude to me.” The Cronbach’s alpha in our study was 0.86.

#### Daily negative emotion

Negative emotion was measured using a scale that consisted of three items, derived from Mackinnon et al.’s (1999) short form of the Positive and Negative Affect Schedule, consistent with previous studies. We selected three discrete negative emotions: anger, nervousness and distress. We chose these three items because they best represented emotions that are negative in hedonic tone and high in intensity (Matta et al., 2014). An example item is “Today, I felt anger.” The internal consistency of negative emotion was 0.89.

#### Daily work engagement

Work engagement was measured using a shortened scale consisting of three items derived from the Utrecht Work Engagement Scale (Schaufeli, 2016). An example item is “Today, I felt strong and vigorous.” The internal consistency of the three items was 0.70.

#### Coworker support

Coworker support was a between-person level variable that was measured only once. Therefore, coworker support was measured using Hammer et al.’s (2004) original scale, which comprises five items. An example item is “I receive help and support from my coworkers,” and the internal consistency of the three items was 0.85.

### Data analysis

For statistical analyses, we used SPSS to calculate descriptive statistics, reliabilities, and correlations, and we used HLM to calculate cross-level regression. In the daily diary research, the same person was surveyed for several days; thus, the data can be seen as two-level data, with each day’s repeated measures (Level 1) nested within individuals (Level 2). We applied HLM to test this model.

## Results

### Preliminary analyses

The means, standard deviations and correlation coefficients of the main variables of this study are shown in Table 1. For the within-person level variables, the correlation coefficients were calculated using day-level variables. Daily abusive supervision had a significant relationship with daily negative emotions (*r* = 0.41, *p* < 0.001) and daily work engagement (*r* = −0.49, *p* < 0.001). Daily negative emotions were significantly related to daily work engagement (*r* = −0.43, *p* < 0.001). The correlation analysis results functioned as a preliminary test of the hypothesis. For the between-person level variables, the correlation coefficients were calculated using person-level variables. The intraclass correlation coefficients of the within-person variables ranged from 0.27 to 0.52, indicating that within-person variability could explain a considerable amount of the variance.

**TABLE 1:** Means, standard deviations, reliability, and correlations.

Variables	M	S	1	2	3	4	5	ICC 1
**Day level**								
1. Abusive supervision	2.64	1.15	(0.86)					27%
2. Negative emotions	2.93	1.50	0.41***	(0.89)				35%
3. Work engagement	3.70	0.93	−0.49***	−0.43***	(0.70)			52%
**Person level**								
4. Coworker support	2.93	1.00	0.04	0.23*	−0.01		(0.85)	

ICC1, intraclass correlation coefficients. *p < 0.05 and ***p < 0.001.

As shown in Table 2, the heterotrait-monotrait (HTMT) ratio of correlations of all variables was less than 0.85; in addition, the square root of average variance extracted of each variable was greater than the correlation coefficients between it and the other variables, indicating discriminative validity among variables. Each construct’s average variance extracted was greater than 0.5, and the composite reliability of all variables was greater than 0.7, indicating that the variables had high convergent validity.

**TABLE 2:** Heterotrait-monotrait (HTMT), AVE, and CR.

Variables	1	2	3	AVE	Square root of AVE	CR
1. Abusive supervision				0.70	0.83	0.92
2. Negative emotions	0.51			0.83	0.91	0.94
3. Work engagement	−0.68	−0.46		0.64	0.81	0.84
4. Coworker support	0.04	0.26	−0.01	0.59	0.78	0.85

HTMT, heterotrait-monotrait ratio; AVE, average variance extracted; CR, composite reliability.

### Multilevel confirmatory factor analysis

We conducted a multilevel confirmatory factor analysis, and the results in Table 3 show that the four-factor model fit the data satisfactorily [χ2 (125) = 211.84, *p* < 0.001, CFI = 0.93, TLI = 0.92, RMSEA = 0.04], surpassing all other alternative models. This indicates that the variables included in this study can be empirically discriminated from each other.

**TABLE 3:** Confirmation factor analysis.

Fitting index	χ^2^	df	RMSEA	CFI	TLI
One-factor model	649.86	134	0.10	0.61	0.53
Two-factor model	522.02	133	0.09	0.71	0.65
Three-factor model	477.19	130	0.09	0.74	0.68
Four-factor model	211.84	125	0.04	0.93	0.92
Four factors + method factor	356.71	126	0.07	0.83	0.78

Four-factor model (AS, NE, WE, CS); Three-factor model (AS + NE, WE, CS); Two-factor model (AS + NE + WE, CS); One-factor model (AS + NE + WE + CS). AS, abusive supervision; NE, negative emotion; WE, work engagement; CS, coworker support.

We used a one-factor test and controlled for the effects of an unmeasured latent methods factor to check for possible common variance (Podsakoff et al., 2012). The one-factor model [χ^2^(134) = 649.86, *p* < 0.001, CFI = 0.61, TLI = 0.53, RMSEA = 0.107] did not reach the statistical requirements (as shown in Table 3), indicating no serious common variance problem in this study. Second, we constructed a latent common method variance factor, allowing all indicators at both the within-person and between-person levels to load on an unmeasured method factor. Then, we developed a five-factor model that includes the four-factor model and CMV. The results in Table 3 show that the five-factor model [χ^2^(126) = 356.71, *p* < 0.001, CFI = 0.83, TLI = 0.78, RMSEA = 0.07] did not fit the data better than the four-factor model. These results suggest that common method variance is not a serious threat to our study.

### Testing of hypotheses

The main effect analysis results are shown in Table 4. First, we built a null model, and then we included the control variables (sex, age, education and trait affect) and predictor variables (abusive supervision). The results show that education (β = 0.13, *p* < 0.01) and trait affect (β = 0.19, *p* < 0.001) had positive relations with daily work engagement and that daily abusive supervision had a significant negative effect on daily work engagement (β = −0.36, *p* < 0.001). Thus, hypothesis 1 is supported.

**TABLE 4:** Multilevel models predicting work engagement.

	Null model	Model 1	Model 2	Model 3	Model 4
					
	Engagement	Emotion	Engagement	Engagement	Emotion
Intercept	3.70***	2.93***	3.71***	3.74***	2.94***
**Control variables**					
Sex		−0.45	−0.07	−0.23^+^	−0.47
Age		−0.28**	−0.07	−0.11^+^	−0.20
Education		−0.05	0.13**	0.12^+^	−0.03
Trait affect		0.25	−0.19***	−0.16***	0.19
**Predictor variables**					
Daily abusive supervision		0.34***	−0.36***	−0.25***	0.34***
Daily negative emotion				−0.25***	
Coworker support					0.19
**Interaction term**					
Abusive supervision × Coworker support					−0.19***
−2 Log (FIML)	867.69	1065.72	788.34	726.66	1057
df	3	10	10	14	12
L1 intercept variance	0.45	0.65	0.41	0.32	0.66
L 2 intercept variance	0.41***	0.99***	0.12***	0.19***	0.92***

**p < 0.01 and ***p < 0.001. ^+^p < 0.1.

We examined the mediating effects of the 1-1-1 model according to Zhang et al.’s (2009) method. The results shown in Table 4 and model 1 indicate that daily abusive supervision was positively related to daily negative emotions (β = 0.34, *p* < 0.001) and negatively related to daily work engagement (β = −0.36, *p* < 0.001). After controlling for daily abusive supervision, daily negative emotions had a significant negative effect on daily work engagement (β = −0.25, *p* < 0.001), and the coefficient of the effect of daily abusive supervision on daily work engagement decreased from −0.36 (*p* < 0.001) to −0.25 (*p* < 0.001). The results indicate that daily negative emotions may partially mediate the relationship between daily abusive supervision and daily work engagement. We also used Preacher and Hayes (2008) bootstrapping procedure to estimate the mediating effect, as shown in Table 5. The confidence intervals for the indirect effects excluded zero. In addition, we conducted multilevel structural equation modeling analyses to assess the mediation effect. The results show that daily abusive supervision has a significant effect on daily negative emotions (β = 0.35, *p* < 0.01), which in turn have a significant effect on daily work engagement (β = −0.33, *p* < 0.001). The indirect effect of daily abusive supervision on daily work engagement *via* daily negative emotions (β = −0.11, *p* < 0.01, 95% CI = −0.18, −0.05) is significant. Therefore, hypothesis 2 is supported.

**TABLE 5:** Bootstrap analyses of indirect effect.

Path	Effect	SE	LLCI	ULCI
Abusive supervision-work engagement	−0.30	0.04	−0.38	−0.23
Abusive supervision-negative emotion-work engagement	−0.10	0.02	−0.13	−0.06

SE, standard error; LLCI, lower level confidence interval; ULCI, upper level confidence interval.

**FIGURE 2:**
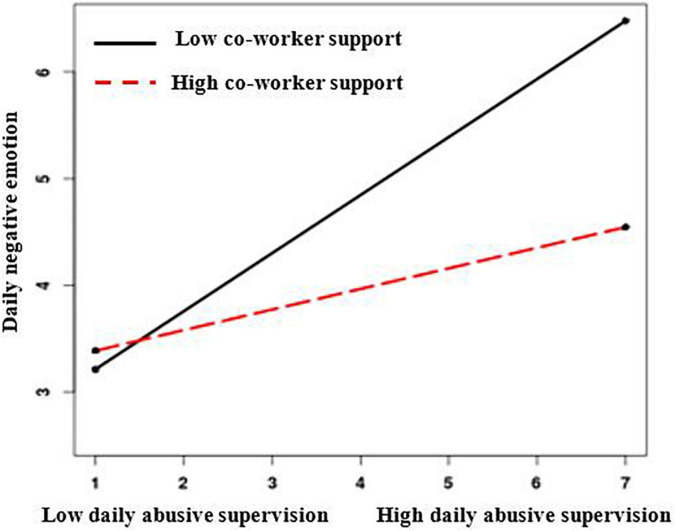
Coworker support as a moderator of the relationship between daily abusive supervision and daily negative emotion.

To test the cross-level moderating effect based on model 1, we built model 4 and included the moderating variable (coworker support) and interaction terms of the independent variable (daily abusive supervision) and the moderating variable (coworker support). The results show that the interaction effect was significant at the 001 level. Moreover, the simple slope results indicate that daily abusive supervision had a positive significant effect on daily negative emotions when employees’ perceived coworker support was low (β = 0.54, *p* < 0.001). No significant relationship was found between daily abusive supervision and daily negative emotions when employees’ perceived coworker support was high (β = 0.19, *p* > 0.05). To improve the interpretability of the interaction effects, we plotted these relations graphically, as shown in Figure 2; therefore, hypothesis 3 is supported.

## Discussion

Consistent with previous research suggesting that a large amount of the variation in abusive supervision and work engagement may be the result of daily changes and fluctuations (Barnes et al., 2015; Marijntje et al., 2020; Zampetakis, 2022), our study finds that approximately 30% of the total variance in abusive supervision and work engagement can be attributed to within-person variation. This finding demonstrates the necessity of using both a within-individual design and a between-individual design to examine the mechanism underlying the effects of abusive supervision on work engagement. At the within-individual level, our study examines the dynamic mechanisms by which daily abusive experience affect daily work engagement. The results show that leaders’ daily abusive supervision is an important daily antecedent of employees’ daily work engagement and indicate that the more abusive behavior employees experience during a day, the less likely they are to be engaged in their work. More specifically, leaders’ abusive behavior directly elicits abused employees’ negative emotions, thus resulting in abused employees having insufficient resources to exhibit work engagement. At the within-individual level, our study finds that coworker support can attenuate the deleterious effect of daily abusive experiences on the daily negative emotions of abused employees’. Specifically, when coworker support is higher, the positive effect of daily abusive experiences on employees’ daily negative emotions is weaker.

### Theoretical contributions

First, on the one hand, extant research lacks a dynamic lens for portraying the influence processes of abusive behaviors (Zhang and Liu, 2018); on the other hand, although we know a great deal regarding the between-level antecedents of work engagement, research concerning day-level predictors of work engagement remains limited (Sonnentag et al., 2020). To help boost work engagement on a daily basis and address the day-to-day dynamics mechanisms of abusive supervision and work engagement, this study employed a daily diary design to explore the dynamic mechanisms of daily abusive supervision affecting daily work engagement through the mediating role of daily negative emotions. This research deepens our understanding of the dynamic formation mechanism of job engagement.

Second, based on COR theory, leadership behavior can both provide resources to employees when it takes the form of constructive leadership behavior and consume employees’ resources when it manifests as destructive leadership behavior. Previous studies have focused on the positive leadership affecting work engagement, such as responsible, authentic, servant, empowering, transformational, inclusive, ethical, and engaging leadership (Breevaart et al., 2014, 2016; Cai et al., 2018; Kirrane et al., 2018; Kaya and Karatepe, 2020; Tuin et al., 2021). Limited research has investigated the effect of leadership on work engagement from the perspective of resource consumption (Venz et al., 2018). However, a growing body of evidence indicates that leaders may engage in destructive leadership (Mackey et al., 2021, p. 705) and that bad is stronger than good (Baumeister et al., 2001). Therefore, it is more important to examine the effect of destructive leader behavior on work engagement. Our study focuses on the effect of abusive supervision, which is a typical and pervasive form of negative leadership in organizations, on work engagement. This study enriches the work engagement research perspective.

Third, COR theory claims that social support, as one of individuals’ most important resources, can increase the available resources that allow the individual to deal with stressors (Arshad et al., 2021). However, the results of extant research regarding the question of whether incongruence between sources of support and stressors is desirable remains ambiguous (Mayo et al., 2012). Our results show that job resources derived from coworker support can significantly alleviate the negative emotions inspired in employees by abusive experiences. When an employee experiences abusive behavior from a leader, support from a coworker has an important moderating effect because in such a situation, support from the leader can cause the abused employee to feel inconsistent and insincere. In addition, our study demonstrates the core idea of the JD-R model, which serve as the main theoretical basis for work engagement research, namely, that job demands (abusive supervision) and job resources (coworker support) have an interaction effect on work engagement (Cooke et al., 2019).

### Practical implications

First, this study suggests that employees’ work engagement fluctuates from day to day and that fluctuation in employees’ abusive experiences is the main factor that determines how engaged employees are in their daily tasks. Therefore, when managers try to solve employee work engagement problems, they should not only look for problems related to employees’ working ability and attitudes but also consider whether managers’ leadership styles are inappropriate. Our results show that an organization can increase employee engagement by reducing supervisors’ abusive behavior. To this end, on the one hand, organizations can help managers establish appropriate concepts through organizational culture and values, causing them to realize that managers are employees’ servicers and that they should respect employees and treat them fairly. On the other hand, it is necessary to strengthen supervisor behavior monitoring and improve bottom-up communication systems and complaint mechanisms so that an organization can detect and reduce managers’ misconduct behaviors.

Second, this study finds that daily abusive behavior affects employees’ daily work engagement mainly through daily negative emotions. Our results suggest that organizations can enhance employees’ emotional knowledge and improve their ability to self-regulate their emotions through emotional management training in management practice. Simultaneously, organizations can enrich communication channels and help employees vent their negative emotions through other channels. In addition, an organization can offer an employee assistance program to help employees regulate negative emotions in a timely manner through professional guidance, training and counseling to prevent negative emotions from spreading throughout the organization. These measures can help an organization build a harmonious emotional atmosphere and thereby decrease the tendency for employees to reduce their negative emotions by reducing work engagement.

Finally, this study shows that coworker support, as an important work resource, can significantly buffer the negative consequences of employees’ abusive experiences. Thus, organizations should pay attention to humanistic care and help employees build harmonious interpersonal relationships to meet their relationship needs. On the one hand, organizations can establish management systems that support employees’ establishment of cooperative relations; for example, organizations should be cautious about using a system of terminating only the lowest-performing employees, which emphasizes performance as the most important factor. On the other hand, an organization can organize interesting group activities to provide opportunities for employee communication and promote workplace friendship. Through the abovementioned measures, employees may feel less isolated and helpless when they experience abusive supervisor behavior because other positive interpersonal interactions could compensate for the negative consequences of abusive experiences.

### Limitations and future research directions

As this study’s first limitation, it used a daily diary survey in which 73 participants completed a survey over 5 consecutive working days, which means that the sample size at the person and day levels may have been insufficient. Previous studies have suggested that the survey period should be no less than 5 days and that the number of participants should be no less than 30 (Ohly et al., 2010). Although our sample size may not lead to biased results, to increase generalizable conclusions and improve statistical power, future studies may have more participants’ complete questionnaires for longer periods of time.

Second, all of the variables of this study were based on self-reports, and all day-level variables were measured at the same time point, which may have increased the potential for common method variance. Abusive supervision, negative emotions and job engagement are all private experiences, and self-reports more closely reflect actual experiences and behaviors (Bakker and Xanthopoulou, 2009). In addition, Demerouti and Cropanzano (2017) pointed out that self-reports should not automatically be viewed as biased. The interaction effect was significant in this study, so the self-report measures did not result in a serious problem or threaten our results. However, to explore causal relationships, a future survey could collect data from separate sources and time points. For example, the participants could complete the questionnaire three times per day; abusive supervision could be measured in the morning, negative emotion could be measured in the afternoon, and work engagement could be measured in the evening.

Third, all participants in this study were Chinese chain restaurant employees; however, chain restaurant employees are characterized by low education, low income, long working hours, young age and other characteristics that make them quite different from employees in other industries. Therefore, whether these conclusions about chain restaurant employees apply to other industries remains to be further demonstrated by scholars. Future studies can explore the reliability of the research conclusions to other industry samples from different regions that take different forms.

## Conclusion

Using a within-individual study design, this study explores the dynamic mechanism of daily abusive supervision on daily work engagement from the perspective of the COR theory. The results show that employees’ daily abusive experiences directly lead to their daily negative emotions and then reduce their daily work engagement. Simultaneously, from the perspective of resources, this paper explores the buffering effect of coworker support on the influence of daily abusive supervision on daily negative emotion. The results show that when coworker support, which is an important job resource, is higher, the adverse effect of employees’ daily abusive supervision on daily negative emotions is weaker.

## Data availability statement

The raw data supporting the conclusions of this article will be made available by the authors, without undue reservation.

## Ethics statement

Ethical review and approval was not required for the study on human participants in accordance with the local legislation and institutional requirements. Written informed consent from the patients/participants was not required to participate in this study in accordance with the national legislation and the institutional requirements.

## Author contributions

HW wrote the original draft of the manuscript and analyzed the data. TT revised the manuscript. Both authors contributed to the design and conceptualization of the manuscript, as well as to reviewing, and editing the manuscript.

## Conflict of interest

The authors declare that the research was conducted in the absence of any commercial or financial relationships that could be construed as a potential conflict of interest.

## Publisher’s note

All claims expressed in this article are solely those of the authors and do not necessarily represent those of their affiliated organizations, or those of the publisher, the editors and the reviewers. Any product that may be evaluated in this article, or claim that may be made by its manufacturer, is not guaranteed or endorsed by the publisher.
